# The impact of different therapies on invasive pulmonary aspergillosis in patients with severe fever and thrombocytopenia syndrome: a systematic review and meta-analysis

**DOI:** 10.3389/fpubh.2026.1834363

**Published:** 2026-07-08

**Authors:** Feng Hao, Yingzi Wang, Siyu Qu, Liquan Zhang, Wenhua Zhang, Lili Wei, Quanman Hu, Na Li, Anmin Ge

**Affiliations:** 1 Yantai Center for Disease Control and Prevention, Yantai, China; 2 Penglai Center for Disease Control and Prevention, Yantai, China; 3 College of Public Health, Zhengzhou University, Zhengzhou, China

**Keywords:** meta-analysis, mortality risk, SAPA, SFTS, therapy

## Abstract

**Background:**

In recent years, a growing number of studies have reported that patients with severe fever with thrombocytopenia syndrome (SFTS)-associated invasive pulmonary aspergillosis (SAPA) patients exhibit a relatively high incidence and mortality rate. However, the conclusions vary considerably and SFTS patients frequently receive immunosuppressive therapies such as corticosteroid therapy, and the influence on the incidence of SAPA remains uncertain. Therefore, this study intends to conduct a comprehensive systematic review and meta-analysis to consolidate existing evidence.

**Methods:**

Effect estimates were expressed as incidence of SAPA, odds ratio (OR) and 95% confidence intervals (CI).

**Results:**

According to the search strategy, a comprehensive search was carried out in the PubMed, Web of Science, and Embase databases. After a detailed assessment, 10 articles met the inclusion criteria and were included in this study. The results indicate that the incidence of SAPA in SFTS patients is 27.4% (95% CI: 18.9–35.9%, I^2^ = 95.60%). The mortality risk among SAPA patients was significantly higher than that among non-SAPA patients: OR = 5.381 (95% CI: 3.666–7.899, I^2^ = 0.00%). Therapies including corticosteroid (OR = 3.104, 95% CI: 2.410–3.998, I^2^ = 35.70%), intravenous immunoglobulin (IVIG) (OR = 3.952, 95% CI: 1.876–8.326, I^2^ = 77.70%), antibiotics (OR = 16.419, 95% CI: 8.489–31.756), and mechanical ventilation (OR = 6.456, 95% CI: 4.362–9.554, I^2^ = 43.80%, *p* = 0.169) are associated with higher odds of SAPA.

**Conclusion:**

Our study indicates that the incidence of SAPA and the associated mortality risk are relatively high. Moreover, therapies including corticosteroids, IVIG, antibiotics, and mechanical ventilation are associated with higher odds of SAPA. Clinicians should maintain a high index of suspicion for SAPA and consider early diagnostic testing and timely antifungal therapy when SAPA is suspected or confirmed.

**Systematic review registration:**

CRD420261343968.

## 1 Introduction

Severe fever with thrombocytopenia syndrome (SFTS) is an endemic tick-borne hemorrhagic fever caused by a novel bunyavirus (SFTSV), which is also known as Dabie bandavirus (DBV) (1). Since the disease was first discovered in China in 2011, it has subsequently been detected in other East Asian countries, including South Korea and Japan (2, 3). Ticks are recognized as vectors of SFTS since the nucleic acid sequences of the SFTSV extracted from ticks exhibit a 95% similarity to those obtained from SFTS patients with a well-documented history of tick bites (4). The incidence of SFTS demonstrates distinct regional clustering, predominantly occurring in regions featuring temperate and humid or subtropical climate characteristics (5). Furthermore, research has indicated that the affected population mainly consists of farmers residing in forest and hilly areas (6). The probable reason is that the climatic factors in these areas, including average temperature, humidity, and sunlight, are conducive to the reproduction of ticks (7).

The incubation period of SFTS is approximately 5 to 14 days and is influenced by the viral load (2). The primary clinical manifestations include a sudden onset of fever accompanied by respiratory or gastrointestinal symptoms, followed by a gradual decrease in platelet count and white blood cell levels (8). The typical clinical course of SFTS is generally divided into three stages: the febrile stage, the multiple organ dysfunction (MOD) stage, and the recovery stage (9). T The febrile stage typically lasts 5 to 11 days. Patients with a high viral load during this stage usually progress to the MOD stage within 3 to 5 days. The MOD stage progresses extremely rapidly and is characterized by hepatic and renal dysfunction, myocardial injury, and coagulopathy (10). The mortality rate among SFTS patients in the MOD stage is exceedingly high, primarily due to excessive inflammatory responses and severe secondary infections leading to multiple organ failure (11). The overall case-fatality rate of SFTS is reported to be 16.2% (95% confidence interval (CI): 14.6–17.8%), posing a substantial threat to public health (12).

In recent years, a growing number of studies have reported that SFTS-associated invasive pulmonary aspergillosis (SAPA) patients, feature a relatively high incidence and mortality rate (13, 14). Invasive pulmonary aspergillosis (IPA) typically affects individuals with weakened immune systems (15). It has been verified that patients with severe SFTS experience immune dysfunction, which accounts for the relatively high incidence of SAPA in secondary infections among these patients. Research indicates that among the common pathogens isolated from secondary infections, nearly 50% are *Aspergillus fumigatus* (16, 17). Although various studies have reported the incidence and mortality incidence of SAPA, the conclusions differ significantly because of disparities in study regions and sample sizes. Moreover, SFTS patients frequently undergo immunosuppressive therapies such as corticosteroid therapy and intravenous immunoglobulin (IVIG) therapy, and the effect of these therapies on the incidence of SAPA remains uncertain. Therefore, this study intends to conduct a comprehensive analysis of the incidence of SAPA, the mortality risk of SAPA, and the impact of treatment on SAPA through a systematic review and meta - analysis, offering a basis for clinical diagnosis and treatment.

## 2 Materials and methods

### 2.1 Literature search methodology

A systematic literature search was conducted in three major academic databases—PubMed, Web of Science, and Embase—to identify all relevant studies published up to March 4, 2026. The search protocol utilizing a carefully constructed combination of keywords and controlled vocabulary terms, including but not limited to: (“Severe Fever with Thrombocytopenia Syndrome” OR “SFTS” OR “SFTSV” OR “Dabie Banda virus”) and (“Invasive Pulmonary Aspergillosis” OR “IPA” OR “Aspergillus”), and the details of the search strategy in different databases can be seen in Supplementary Table S1.

A systematic search was conducted to identify existing reviews and meta-analyses related to the topic; no duplicate or overlapping publications were found. Subsequently, titles and abstracts of retrieved records were screened for relevance, and full-text articles meeting the inclusion criteria were obtained.

### 2.2 Inclusion and exclusion criteria

Inclusion criteria were defined as follows: (1) Studies reporting the incidence of SAPA; (2) Studies examining the association between SAPA and mortality; (3) Studies evaluating how different therapies influence the SAPA incidence.

Exclusion criteria were defined as follows: (1) review articles, corrigenda, and editorial comments; (2) The study did not clearly report the required data such as the SAPA incidence, etc.

### 2.3 Study selection and data extraction

This review followed the PRISMA 2020 statement (registration ID: CRD420261343968) for systematic reporting (18), and the PICOS framework c can be found in Supplementary Table S2. Two reviewers independently assessed records retrieved from electronic databases against prespecified eligibility criteria. Discrepancies in screening decisions were resolved through discussion; when consensus could not be reached, a third reviewer adjudicated. From eligible studies, the following data were extracted: lead author, geographic location of the study, publication or data collection period, mean participant age, and proportion of male participants. Study quality was appraised using the Newcastle–Ottawa Scale (NOS). Studies scoring ≥7 points were classified as high quality; those scoring 5–6 points as moderate quality; and those scoring ≤4 points as low quality (19).

### 2.4 Statistical analysis

Statistical analyses were performed using STATA version 12.1 (Stata Corp, College Station, TX, USA). A two-sided significance threshold of *p* < 0.05 was applied throughout. Effect estimates included incidence rates, odds ratio (OR), and corresponding 95% CI. Heterogeneity across studies was quantified using the I^2^ statistic; values exceeding 50% or *p* < 0.05 in the Cochran’s Q test indicated substantial heterogeneity. In such instances, a random-effects model was adopted to account for both within-study and between-study variability. When heterogeneity was not statistically significant, a fixed-effects model was used to compute pooled estimates (20). Subgroup analyses were conducted according to prespecified characteristics—including population, study period, male %, mean age and epidemiologically relevant covariates—to explore potential sources of heterogeneity. Publication bias was evaluated visually via funnel plots and formally tested using Begg’s rank correlation test and Egger’s linear regression test. Finally, sensitivity analyses—based on sequential omission of individual studies—were carried out to examine the robustness and stability of the overall findings.

## 3 Results

### 3.1 Study selection

**Figure 1:**
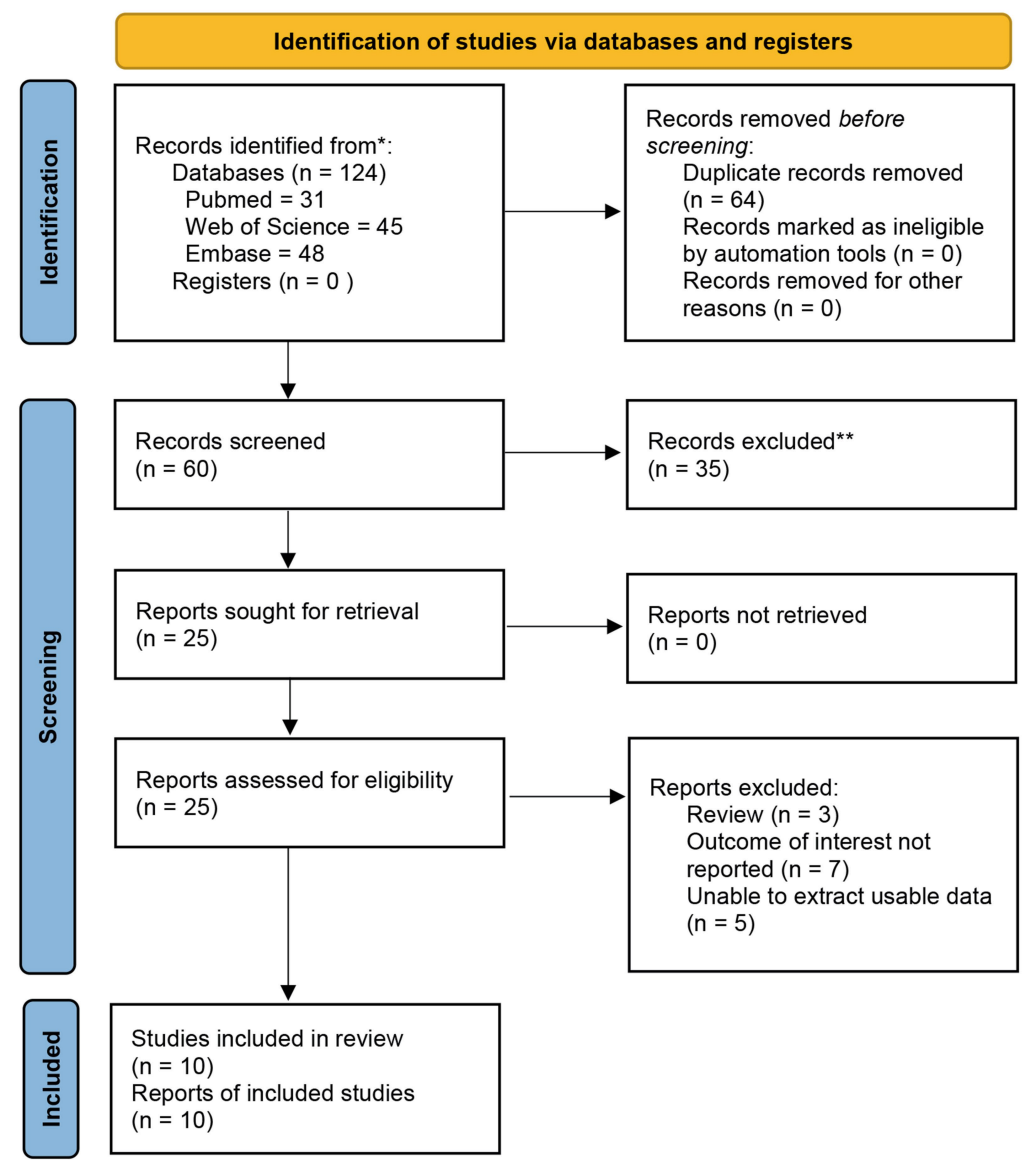
Flowchart showing the screening process for included articles.

A systematic search was conducted in PubMed, Web of Science, and Embase up to 4 March 2026. A total of 124 articles were retrieved. After removing duplicates, 60 articles were remained for further screening. Subsequently, 35 articles were excluded during title and abstract screening, and the remaining 25 articles were subjected to full-text evaluation. After a detailed assessment, a total of 10 articles satisfied the predefined eligibility criteria and were consequently selected for inclusion in this analysis (Figure 1) (14, 21–29). All 10 included articles reported the incidence of SAPA, and 8 articles reported the impact of SAPA on the mortality risk. The effects of different therapies on the incidence of SAPA mainly involved corticosteroid therapy (5 articles), IVIG therapy (4 articles), ribavirin therapy (3 articles), antibiotics therapy (4 articles), and mechanical ventilation (3 articles).

Among the included studies, as presented in Table 1, the incidence of SAPA ranged from a minimum of 10.23% to a maximum of 43.87%. The study areas were predominantly in China, with only one study conducted in South Korea. The mean age of SFTS patients included in these studies was all above 50 years old, and the study sample size varied from 45 to 1,650. Regarding the male%, there were 7 studies where the male percentage exceeded 50%. Finally, there was the NOS Score. Only one study had a score of 6 points, and the rest all had scores of 7 points or higher. The specific scoring criteria can be found in Supplementary Table S3.

**Table 1:** Characteristics of the included studies on SAPA.

Author	Study location	Study period	Mean age	Population	Male %	SAPA%	NOS Score	Diagnosing SAPA
Bae et al. (21)	Seoul, South Korea	2014–2018	61.7	45	60	20.00	6	the putative aspergillosis definition^*2^
Dai et al. (22)	Nanjing, China	2011–2021	63.16	189	52.9	20.63	8	EORTC/MSG^*1^ (proven and probable)
Hu et al. (7)	Anhui, China	2015–2019	66.75	76	60.5	39.47	7	EORTC/MSG^*1^ (proven and probable)
Huang et al. (24)	Shandong, China	2021–2024	63.4	96	56.2	36.46	8	EORTC/MSG^*1^ (proven and probable)
Song et al. (25)	Shandong, China	2021–2022	64.7	67	50.7	32.84	8	EORTC/MSG^*1^
Wang et al. (26)	Wuhan, China	2017–2022	65.71	269	62.08	43.87	7	EORTC/MSG^*1^ (probable)
Xu et al. (14)	Nanjing, China	2016–2022	62.68	99	51.5	21.21	8	EORTC/MSG^*1^ (proven)
Xu et al. (27)	Nanjing, China	2016–2019	62	91	41.8	31.87	7	EORTC/MSG^*1^ (proven and probable)
Yan et al. (28)	Nanjing, China	2016–2024	67	360	48.9	21.67	8	EORTC/MSG^*1^ (proven)
Yao et al. (29)	Hubei and Anhui, China	2013–2022	61	1,650	44.8	10.23	9	An IPA diagnosis was reached when any of the subsequent criteria were met, irrespective of host factors or clinical attribute ^*3^

SAPA, SFTS-associated invasive pulmonary aspergillosis; EORTC/MSG, European Organization for the Research and Treatment of Cancer/Mycosis Study Group. *1: (i) Proven SAPA: histopathological evidence of hyphal invasion with tissue damage in lung/bronchial biopsies; (ii) Probable SAPA (requiring all criteria): compatible respiratory symptoms, characteristic chest CT abnormalities and plus one mycological indicator (Aspergillus isolation from sputum, endotracheal aspirate, or BALF; elevated galactomannan (GM) index: serum/plasma: ≥ 1.0 (single sample); BALF: ≥ 1.0; or serum/plasma: ≥ 0.7 and BALF: ≥ 0.8). *2: the putative aspergillosis definition was based on clinical, radiological, and mycological criteria as the presence of the following 3 criteria: compatible signs and symptoms of IPA, abnormal findings on either a chest X ray or CT scan of the lungs, and mycological criteria such as histopathologic evidence of hyphae with recovery of Aspergillus from tissue, positive culture from a bronchoalveolar lavage (BAL), galactomannan (GM) index in BAL ≥ 1, or GM index in serum ≥0.5. Two independent radiologists analyzed the radiological findings of patients classified with PIPA through review of chest X rays and CT images. *2: The proven diagnosis was based on the following criteria: (1) microscopic examination of the samples proved the growth of Aspergillus hyphae, (2) the positive culture of Aspergillus spp. from a normally sterile and clinically or radiologically abnormal site, excluding bronchoalveolar lavage (BAL) fluid, and (3) amplifying the Aspergillus DNA and sequencing the PCR-amplified products. The diagnosis was considered probable if the patients have appropriate host factors, clinical manifestations, and mycological evidence. *3: IPA diagnosis was reached when any of the subsequent criteria were met, irrespective of host factors or clinical attributes: the emergence of unexplained cavities on a chest computed tomography (CT) scan, a galactomannan (GM) index ≥1.0 in bronchoalveolar lavage fluid (BALF), serum GM index>0.5, positive aspergillus culture from BALF, or the confluence of a positive aspergillus culture from tracheal aspirate and a GM index for BALF ranging between 0.5 and 1.0.

### 3.2 The overall effect and 95% CI of different variables

**Figure 2:**
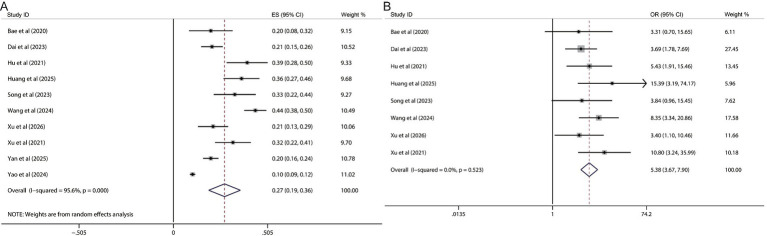
Forest plot showing the overall effect and 95% CI of SAPA incidence **(A)**, mortality risk **(B)**.

Firstly, we analyzed the overall incidence of SAPA, and the results are shown in Figure 2A. The overall incidence of SAPA is 27.4% (95% CI: 18.9–35.9%, I^2^ = 95.60%, *p* < 0.01). The mortality risk of SAPA patients was significantly higher than that in non - SAPA patients: OR = 5.381 (95% CI: 3.666–7.899, I^2^ = 0.00%, *p* = 0.523, Figure 2B).

**Figure 3:**
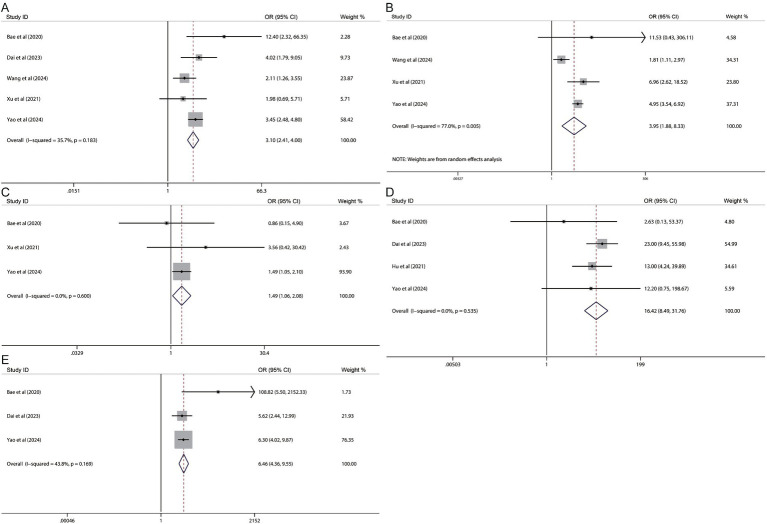
Forest plot showing the overall OR and 95% CI of corticosteroid therapy **(A)**, IVIG therapy **(B)**, ribavirin therapy **(C)**, antibiotics therapy **(D)** and mechanical ventilation **(E)**.

Additionally, in order to exclude one of the potentially overlapping cohorts, we removed the study by Xu et al., and the results are presented in Supplementary Figure S1. The overall incidence of SAPA among the remaining studies was 26.9% (95% CI: 17.9–35.8%, I^2^ = 95.80%, *p* < 0.01), and the mortality risk of SAPA patients was also higher than non - SAPA patients, with an OR of 4.482 (95% CI: 3.001–6.694, I^2^ = 0.00%, *p* = 0.561). Finally, we investigated the effects of different therapies on SAPA: corticosteroid therapy (OR = 3.104, 95% CI: 2.410–3.998, I^2^ = 35.70%, *p* = 0.183, Figure 3A), IVIG therapy (OR = 3.952, 95% CI: 1.876–8.326, I^2^ = 77.70%, *p* = 0.005, Figure 3B), ribavirin therapy (OR = 1.487, 95% CI: 1.065–2.076, I^2^ = 0.00%, *p* = 0.600, Figure 3C), antibiotics therapy (OR = 16.419, 95% CI: 8.489–31.756, I^2^ = 0.00%, *p* = 0.535, Figure 3D), and mechanical ventilation (OR = 6.456, 95% CI: 4.362–9.554, I^2^ = 43.80%, *p* = 0.169, Figure 3). In addition, other therapies such as antifungal therapy, vasopressor therapy, and renal replacement therapy were not comprehensively analyzed due to the insufficient number of included studies.

### 3.3 Subgroup analysis

We conducted subgroup analyses on factors with high heterogeneity (the incidence of SAPA and IVIG therapy) according to the grouping criteria of population, study period, male %, and mean age. Subgroup analyses were carried out for each factor separately. Owing to the limited number of included studies, a binary classification method was employed to categorize these grouping bases.

Regarding the incidence of SAPA, the heterogeneity among studies with certain similar basic characteristics was relatively low. For example, the overall incidence of SAPA for studies with the population <= 100 was 30% (95% CI: 24–37%, I^2^ = 59.5%, *p* = 0.030, Supplementary Figure S2A). Similarly, for studies with the study period <= 4, the overall incidence of SAPA was 32% (95% CI: 26–39%, I^2^ = 39.5%, *p* = 0.158, Supplementary Figure S2B). However, no studies with low heterogeneity were found in relation to the male% and mean age. In the case of IVIG therapy, the male% factor was a source of heterogeneity. The overall OR for studies with the male% > 50% was 2.18 (95% CI: 0.76–6.50, I^2^ = 16.5%, *p* = 0.274, Supplementary Figure S3C), whereas for studies with the male% < = 50%, the overall OR was 5.13 (95% CI: 3.74–7.04, I^2^ = 0.00%, *p* = 0.518, Supplementary Figure S3C). Moreover, for studies with the mean age <= 64, the overall OR was 5.17 (95% CI: 3.77–7.09, I^2^ = 0.00%, *p* = 0.723, Supplementary Figure S3D). Finally, although the heterogeneity among studies with the population <= 100 (Supplementary Figure S3A) and the study period <= 4 (Supplementary Figure S3B) was relatively low, no source of heterogeneity was identified.

### 3.4 Assessment of publication bias and sensitivity analysis

**Figure 4:**
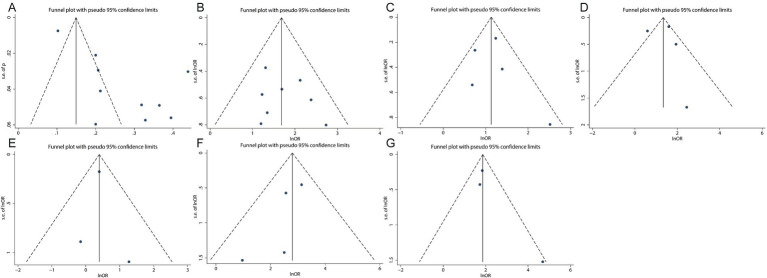
Funnel plot on incidence of SAPA **(A)**, mortality risk **(B)**, corticosteroid therapy **(C)**, IVIG therapy **(D)**, ribavirin therapy **(E)**, antibiotics therapy **(F)** and mechanical ventilation **(G)**.

We examined potential publication bias affecting the pooled effect estimate and its 95% CI across included factors. Funnel plot visualization, along with Begg’s and Egger’s tests, were used for this evaluation (Figure 4). The funnel plot reveals a symmetrical distribution of the overall effects and 95% CI for the incidence of SAPA, mortality risk, corticosteroid therapy, IVIG therapy, ribavirin therapy, antibiotics therapy, and mechanical ventilation. This indicates that there is no obvious publication bias. Similarly, the results of the Begg’s test demonstrated that the *p*-values of all factors were greater than 0.05 (Supplementary Table S4), suggesting that there was no statistically significant small - sample effect. However, it is worth noting that there may be publication bias in the Egger’s test for the incidence of SAPA e (t = 0.419, *p* = 0.033, Supplementary Table S4). Overall, the results suggest that the estimated incidence of SAPA e, case fatality risk, and the influence of therapies on incidence of SAPA are unlikely to be substantially affected by severe publication bias, and the 2 × 2 raw data of the included studies can be seen in Supplementary Table S5.

**Figure 5:**
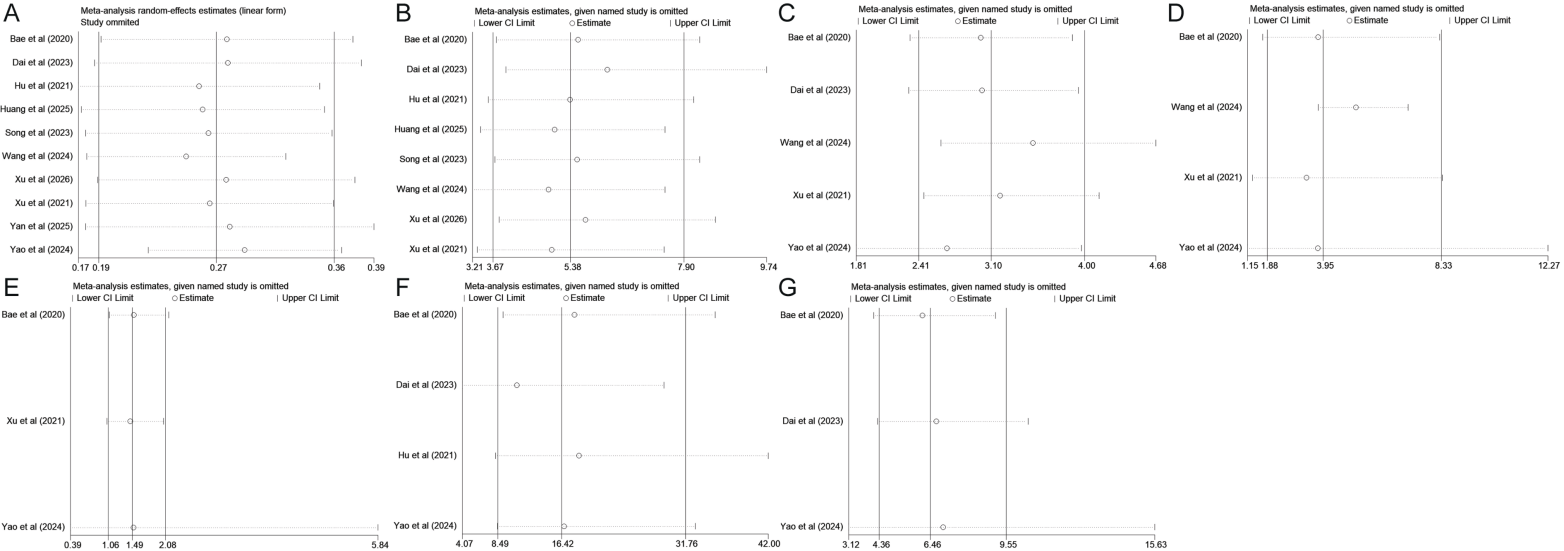
Sensitivity analysis on incidence of SAPA **(A)**, mortality risk **(B)**, corticosteroid therapy **(C)**, IVIG therapy **(D)**, ribavirin therapy **(E)**, antibiotics therapy **(F)** and mechanical ventilation **(G)**.

Subsequently, sensitivity analyses were performed, and by removing each study individually, it was examined whether the combined estimates of the remaining studies were consistent with the population. The results, including the incidence of SAPA, mortality risk of SAPA, the effects of different therapies on the incidence of SAPA involved corticosteroid therapy, IVIG therapy, antibiotics therapy, and mechanical ventilation demonstrated the stability and reliability of the study results (Figures 5A–D, F, G). However, the results for ribavirin therapy showed instability (Figure 5E).

## 4 Discussion

IPA is a severe opportunistic infection that occurs as a secondary consequence of immunosuppression or immune dysfunction. It is more prevalent among patients with immunosuppressive conditions (e.g., agranulocytosis), those receiving high-dose corticosteroid and immunosuppressant therapy, and those with organ transplants or acquired immune deficiency syndrome (AIDS); it carries a relatively high mortality rate (30, 31). It is well established that leukopenia is a key risk factor for aspergillosis, as it impairs both innate and adaptive immunity (32). Therefore, these factors account for the higher odds of SAPA.

Given the varying SAPA incidence rates and SAPA-associated mortality risks across different studies, we conducted a comprehensive systematic review and meta-analysis. First, we estimated the pooled SAPA incidence rate across all included studies, which was 27.4% (95% CI: 18.9–35.9%)—substantially higher than that observed in non-immunocompromised influenza patients (14%) (33). It is worth noting that although the sensitivity analysis results indicated stable outcomes, the heterogeneity among different studies was relatively high. Subgroup analysis showed that among studies with the population of < =100 and the study period of < =4, the heterogeneity was relatively low. The possible reason is that the time span of these studies was short, and the update of clinical treatment guidelines within a short period would not cause significant changes. Therefore, the incidence of SAPA did not change much. In addition, there are differences among studies in terms of diagnostic criteria and inclusion populations. Some studies included both proven and probable SAPA cases, whereas others included only proven SAPA cases. Moreover, cohort overlap or variations in disease severity, ICU admission rates, and antifungal prophylaxis across different studies may account for the high heterogeneity observed in the pooled SAPA incidence rates. Further comprehensive analysis revealed that SAPA patients had a higher mortality risk compared to non-SAPA patients, with virtually no heterogeneity across the included studies. Some studies suggest that Aspergillus infection is associated with increased organ damage (15). And multiple organ functional damage such as liver and kidney damage is an important cause of death in SFTS.

Then, we analyzed common treatment for the risk of SAPA. First, we found that corticosteroid therapy is associated with increased odds of SAPA. Dai et al.’s study shows that after adjusting for potential confounding variables, the use of corticosteroid elevates the risk of SAPA (22). The possible reason is that the use of corticosteroid not only reduces the activity and phagocytic function of white blood cells but also alleviates the inhibitory effect of lung macrophages on the growth of Aspergillus (34, 35). Moreover, a retrospective analysis of propensity score matching in Japan indicates that the use of corticosteroid therapy significantly raises the incidence of secondary infections, especially SAPA, and should be carefully considered in clinical practice (36). In addition, after infection, patients with SFTS not only experience suppression of innate immunity, manifested as a decrease in monocyte count (37), but also sustain damage to adaptive immunity, specifically reflected in significant reductions in both the number and functional activity of CD3^+^ and CD4^+^ T lymphocytes (38). Therefore, corticosteroid therapy may have aggravated these immunosuppressive states, thereby increasing the incidence of SAPA.

IVIG is a blood product prepared from the pooled plasma of healthy donors and contains a high concentration of IgG. ue to its anti-inflammatory properties, it has been used to treat various viral infections. However, a 10-year study evaluating IVIG for the treatment of SFTS found no significant benefit in reducing mortality or improving clinical prognosis (39). Additionally, Zuo et al.’s study on invasive pulmonary fungal infection (IPFI) in SFTS patients revealed that IVIG therapy was associated with increased odds IPFI: OR = 3.270 (95% CI: 1.424–7.580, *p* = 0.005) (40). The mechanism underlying this association remains unclear. Notably, IVIG is prepared from pooled plasma of thousands of donors, and in SFTSV-endemic areas it may contain anti-SFTSV antibodies. Therefore, the elevated IPFI is unlikely to be attributable to a simple lack of virus-specific antibodies in the formulation. It may rather involve the complex immunomodulatory effects of IVIG or the profound immunosuppression caused by SFTSV itself.

For antibiotics therapy, in clinical practice, when severe SFTS patients develop secondary infections, bacterial infections are typically considered. Thus, antibacterial therapy (most are antibiotics) is initially employed for the treatment of secondary infections. Hu et al.’s research revealed that patients with severe SFTS may also be infected with Aspergillus in the early stage of the disease (23). Therefore, failure to administer antifungal therapy promptly upon secondary infection increases the incidence of SAPA. However, a six-year SAPA study indicated that the difference in antifungal therapy between the SAPA group and the non-SAPA group was not statistically significant (26). Meanwhile, exposure to broad-spectrum antibiotics has been identified as an independent risk factor for invasive pulmonary aspergillosis, likely through disruption of the intestinal microbiota and impairment of colonization resistance against fungi (41). Moreover, a study on the treatment of SAPA with voriconazole (VRZ) suggested that antifungal therapy could significantly reduce the incidence of SAPA, ICU admission rates, and mortality (24). Mechanical ventilation is commonly used as an effective therapy for dyspnea in SFTS patients. This study demonstrates that this respiratory support heightens the risk of SAPA. In the study of COVID-19-associated pulmonary aspergillosis (CAPA), respiratory support was also identified as a risk factor (42), possibly because dyspnea may damage epithelial cells and augment the risk of fungal infection. In addition, these associations must be viewed cautiously. Antibiotics therapy and mechanical ventilation were most plausibly markers of disease severity in the included studies, because sicker patients are simultaneously more likely to receive these interventions and to develop SAPA. Because of inadequate adjustment for confounding, these results should be interpreted with caution. Finally, there is the impact of ribavirin therapy on SAPA. It is worth noting that the results of the sensitivity analysis are unstable. Generally, a high viral load is considered one of the indicators of poor prognosis for SFTS (43, 44). Wang et al.’s study revealed that the initial viral load in the SAPA group was higher than that in the non-SAPA group (26). However, there is currently a lack of mechanistic study on how the viral load level affects SAPA. Therefore, the impact of ribavirin on the incidence of SAPA may be achieved by influencing viral replication in patients. Nevertheless, a meta analysis of ribavirin in the treatment of SFTS indicated that ribavirin did not have a positive effect on the treatment of SFTS and was only effective in reducing viral replication for patients with a low viral load (45).

This study also has certain limitations. First, insufficient research has been conducted on some other therapeutic approaches—such as platelet (PLT) transfusion, vasopressor use, and renal replacement therapy—to allow for a comprehensive analysis. Second, the sample sizes of some included studies were too small, resulting in excessively wide confidence intervals and thereby compromising the robustness of the findings. Third, two of the included studies originated from the same medical center in Nanjing. Although the time periods of these studies differed, we could not fully rule out the possibility of participant overlap. Finally, the majority of the included studies were conducted in China, with limited representation from other countries or regions.

## 5 Conclusion

This study conducted a comprehensive systematic review and meta-analysis to examine the incidence of SAPA in SFTS patients, the associated mortality risk, and the impact of various therapeutic interventions on SAPA. The results indicate that the incidence of SAPA is 27.4%, and the associated mortality risk is relatively high. Certain therapies—such as corticosteroid therapy, IVIG therapy, antibiotic therapy, and mechanical ventilation—are associated with higher odds of SAPA. Clinicians should maintain a high index of suspicion for SAPA and consider early diagnostic testing and timely antifungal therapy when SAPA is suspected or confirmed.

## Data Availability

The original contributions presented in the study are included in the article/Supplementary material, further inquiries can be directed to the corresponding authors.

## References

[1] Yu XJ Liang MF Zhang SY Liu Y Li JD Sun YL . Fever with thrombocytopenia associated with a novel Bunyavirus in China. N Engl J Med. (2011) 364:1523–32. doi: 10.1056/NEJMoa1010095, 21410387 PMC3113718

[2] Liu Q He B Huang SY Wei F Zhu XQ. Severe fever with thrombocytopenia syndrome, an emerging tick-borne zoonosis. Lancet Infect Dis. (2014) 14:763–72. doi: 10.1016/s1473-3099(14)70718-2, 24837566

[3] Zhan J Wang Q Cheng J Hu B Li J Zhan F . Current status of severe fever with thrombocytopenia syndrome in China. Virol Sin. (2017) 32:51–62. doi: 10.1007/s12250-016-3931-1, 28251515 PMC6598917

[4] Sun H Hu Q Lu S Yang Y Zhang L Long J . Current status of severe fever with thrombocytopenia syndrome in China (review). Int J Mol Med. (2025) 56:1–17. doi: 10.3892/ijmm.2025.5610, 40849814 PMC12373438

[5] Miao D Liu MJ Wang YX Ren X Lu QB Zhao GP . Epidemiology and ecology of severe fever with thrombocytopenia syndrome in China, 2010–2018. Clin Infect Dis. (2021) 73:e3851–8. doi: 10.1093/cid/ciaa1561, 33068430 PMC8664468

[6] Du Z Wang Z Liu Y Wang H Xue F Liu Y. Ecological niche modeling for predicting the potential risk areas of severe fever with thrombocytopenia syndrome. Int J Infect Dis. (2014) 26:1–8. doi: 10.1016/j.ijid.2014.04.006, 24981427

[7] Hu Q Hu Y Yang Y Chen J Zhang S Zhao F . Short-term effects of meteorological factors on severe fever with thrombocytopenia syndrome incidence in Xinyang, China. Geohealth. (2025) 9:e2025GH001440. doi: 10.1029/2025gh001440, 40765641 PMC12320122

[8] Peng C Hao Y Yuan Y Ma W Zhang D Kong J . Bandavirus Dabieense: a review of epidemiology, clinical characteristics, pathophysiology, treatment and prevention. Virulence. (2025) 16:2520343. doi: 10.1080/21505594.2025.2520343, 40519175 PMC12184167

[9] Yuan F Zheng A. Entry of severe fever with thrombocytopenia syndrome virus. Virol Sin. (2017) 32:44–50. doi: 10.1007/s12250-016-3858-6, 27995422 PMC6598886

[10] Zhang S Wang J Zhang Q Pan Y Zhang Z Geng Y . Association of Liver Function and Prognosis in patients with severe fever with thrombocytopenia syndrome. PLoS Negl Trop Dis. (2024) 18:e0012068. doi: 10.1371/journal.pntd.0012068, 38626222 PMC11051684

[11] Zhang Y Huang Y Xu Y. Associated microbiota and treatment of severe fever with thrombocytopenia syndrome complicated with infections. J Med Virol. (2022) 94:5916–21. doi: 10.1002/jmv.28059, 35945160

[12] Li H Lu QB Xing B Zhang SF Liu K Du J . Epidemiological and clinical features of laboratory-diagnosed severe fever with thrombocytopenia syndrome in China, 2011-17: a prospective observational study. Lancet Infect Dis. (2018) 18:1127–37. doi: 10.1016/s1473-3099(18)30293-7, 30054190

[13] Chen X Yu Z Qian Y Dong D Hao Y Liu N . Clinical features of fatal severe fever with thrombocytopenia syndrome that is complicated by invasive pulmonary Aspergillosis. J Infect Chemother. (2018) 24:422–7. doi: 10.1016/j.jiac.2018.01.005, 29428567

[14] Xu Y Liu Y Qian Y Liu N Tang J Dong D . Characteristics of T-lymphocyte subsets in patients with severe fever with thrombocytopenia syndrome complicated with invasive pulmonary Aspergillosis: a retrospective study. Front Immunol. (2025) 16:1748830. doi: 10.3389/fimmu.2025.1748830, 41660614 PMC12876148

[15] Heylen J Vanbiervliet Y Maertens J Rijnders B Wauters J. Acute invasive pulmonary Aspergillosis: clinical presentation and treatment. Semin Respir Crit Care Med. (2024) 45:069–87. doi: 10.1055/s-0043-1777769, 38211628

[16] Song H Zou S Huang Y Wang Y Wang T Wei W . The pathogenic and clinical characteristics of severe fever with thrombocytopenia syndrome patients with co-infections. Front Cell Infect Microbiol. (2023) 13:1298050. doi: 10.3389/fcimb.2023.1298050, 38106473 PMC10722497

[17] Sun SH Liu RN Zhang SJ Wang ZX. Risk factors for co-infections in patients with severe fever with thrombocytopenia syndrome. J Med Virol. (2025) 97:e70175. doi: 10.1002/jmv.70175, 39817591

[18] Page MJ Moher D Bossuyt PM Boutron I Hoffmann TC Mulrow CD . Prisma 2020 explanation and elaboration: updated guidance and exemplars for reporting systematic reviews. BMJ. (2021) 372:n160. doi: 10.1136/bmj.n160, 33781993 PMC8005925

[19] Lo CK Mertz D Loeb M. Newcastle-Ottawa scale: comparing reviewers' to authors' assessments. BMC Med Res Methodol. (2014) 14:45. doi: 10.1186/1471-2288-14-45 24690082 PMC4021422

[20] Borenstein M Hedges LV Higgins JP Rothstein HR. A basic introduction to fixed-effect and random-effects models for Meta-analysis. Res Synth Methods. (2010) 1:97–111. doi: 10.1002/jrsm.12, 26061376

[21] Bae S Hwang HJ Kim MY Kim MJ Chong YP Lee SO . Invasive pulmonary Aspergillosis in patients with severe fever with thrombocytopenia syndrome. Clin Infect Dis. (2020) 70:1491–4. doi: 10.1093/cid/ciz673, 31342053

[22] Dai Y Pu Q Hu N Zhu J Han Y Shi P . The dose-response relationship between smoking and the risk factor for invasive pulmonary Aspergillosis in patients with severe fever with thrombocytopenia syndrome. Front Microbiol. (2023) 14:1209705. doi: 10.3389/fmicb.2023.1209705, 37455744 PMC10348827

[23] Hu L Kong Q Yue C Xu X Xia L Bian T . Early-warning immune predictors for invasive pulmonary Aspergillosis in severe patients with severe fever with thrombocytopenia syndrome. Front Immunol. (2021) 12:576640. doi: 10.3389/fimmu.2021.576640, 34025635 PMC8138034

[24] Huang D Song L Zou W Wang L Sai L. Evaluation of the efficacy of prophylactic use of Voriconazole against invasive pulmonary Aspergillosis among severe fever with thrombocytopenia syndrome patients in China. J Med Virol. (2025) 97:e70500. doi: 10.1002/jmv.70500, 40673711

[25] Song L Zhao Y Wang G Zou W Sai L. Investigation of predictors for invasive pulmonary Aspergillosis in patients with severe fever with thrombocytopenia syndrome. Sci Rep. (2023) 13:1538. doi: 10.1038/s41598-023-28851-2, 36707667 PMC9883384

[26] Wang H Luo M Fisher D Pronyuk K Musabaev E Thu HNT . Clinical factors associated with invasive pulmonary Aspergillosis in patients with severe fever with thrombocytopenia syndrome: analysis of a 6-year clinical experience. Front Microbiol. (2024) 15:1448710. doi: 10.3389/fmicb.2024.1448710, 39328917 PMC11424530

[27] Xu Y Shao M Liu N Tang J Gu Q Dong D. Invasive pulmonary Aspergillosis is a frequent complication in patients with severe fever with thrombocytopenia syndrome: a retrospective study. Int J Infect Dis. (2021) 105:646–52. doi: 10.1016/j.ijid.2021.02.088, 33640568

[28] Yan R Cao K Li T Qi H Liu Y Qian Y . Establishment and validation of a nomogram for predicting severe fever with thrombocytopenia syndrome complicated by invasive pulmonary Aspergillosis. BMC Infect Dis. (2025) 25:1783. doi: 10.1186/s12879-025-12218-1, 41275152 PMC12751470

[29] Yao L Shi Y Fu J Fang X Zhang H Luo D . Risk factors for invasive pulmonary Aspergillosis in patients with severe fever with thrombocytopenia syndrome: a multicenter retrospective study. J Med Virol. (2024) 96:e29647. doi: 10.1002/jmv.29647, 38708790

[30] Rahi MS Jindal V Pednekar P Parekh J Gunasekaran K Sharma S . Fungal infections in hematopoietic stem-cell transplant patients: a review of epidemiology, diagnosis, and management. Ther Adv Infect Dis. (2021) 8:20499361211039050. doi: 10.1177/20499361211039050, 34434551 PMC8381463

[31] Thornton CR. Breaking the Mould - novel diagnostic and therapeutic strategies for invasive pulmonary Aspergillosis in the immune deficient patient. Expert Rev Clin Immunol. (2014) 10:771–80. doi: 10.1586/1744666x.2014.904747, 24689528

[32] Evangelidis P Tragiannidis K Vyzantiadis A Evangelidis N Kalmoukos P Vyzantiadis TA . Invasive fungal disease after chimeric antigen receptor-T immunotherapy in adult and pediatric patients. Pathogens. (2025) 14:170. doi: 10.3390/pathogens14020170, 40005545 PMC11858289

[33] Schauwvlieghe A Rijnders BJA Philips N Verwijs R Vanderbeke L Van Tienen C . Invasive aspergillosis in patients admitted to the intensive care unit with severe influenza: a retrospective cohort study. Lancet Respir Med. (2018) 6:782–92. doi: 10.1016/s2213-2600(18)30274-1, 30076119

[34] Jung SI Kim YE Yun NR Kim CM Kim DM Han MA . Effects of steroid therapy in patients with severe fever with thrombocytopenia syndrome: a multicenter clinical cohort study. PLoS Negl Trop Dis. (2021) 15:e0009128. doi: 10.1371/journal.pntd.0009128, 33606699 PMC7928499

[35] White PL Dhillon R Cordey A Hughes H Faggian F Soni S . A National Strategy to diagnose coronavirus disease 2019-associated invasive fungal disease in the intensive care unit. Clin Infect Dis. (2021) 73:e1634–44. doi: 10.1093/cid/ciaa1298, 32860682 PMC7499527

[36] Kawaguchi T Umekita K Yamanaka A Hara S Yamaguchi T Inoue E . Corticosteroids May have negative effects on the Management of Patients with severe fever with thrombocytopenia syndrome: a case-control study. Viruses. (2021) 13:785. doi: 10.3390/v13050785, 33925061 PMC8145003

[37] Peng C Wang H Zhang W Zheng X Tong Q Jie S . Decreased monocyte subsets and Tlr4-mediated functions in patients with acute severe fever with thrombocytopenia syndrome (Sfts). Int J Infect Dis. (2016) 43:37–42. doi: 10.1016/j.ijid.2015.12.009, 26701820

[38] Sun L Hu Y Niyonsaba A Tong Q Lu L Li H . Detection and evaluation of Immunofunction of patients with severe fever with thrombocytopenia syndrome. Clin Exp Med. (2014) 14:389–95. doi: 10.1007/s10238-013-0259-0, 24068614 PMC7101760

[39] Zhang SS Du J Cui N Yang X Zhang L Zhang WX . Clinical efficacy of immunoglobulin on the treatment of severe fever with thrombocytopenia syndrome: a retrospective cohort study. EBioMedicine. (2023) 96:104807. doi: 10.1016/j.ebiom.2023.104807, 37738834 PMC10520313

[40] Zuo Y Wang H Huang J Zhang F Lv D Meng T . Pulmonary infection in patients with severe fever with thrombocytopenia syndrome: a multicentre observational study. J Med Virol. (2023) 95:e28712. doi: 10.1002/jmv.28712 36991571

[41] Liu F Wang Z Hai Y Wu H Yang Y Chen W . Risk and prognostic patterns of viral-associated invasive pulmonary Aspergillosis: impact of corticosteroid and antibiotic exposure in non-neutropenic hosts. J Thorac Dis. (2025) 17:8030–45. doi: 10.21037/jtd-2025-1198, 41229807 PMC12603457

[42] Montrucchio G Lupia T Lombardo D Stroffolini G Corcione S De Rosa FG . Risk factors for invasive aspergillosis in ICU patients with COVID-19: current insights and new key elements. Ann Intensive Care. (2021) 11:136. doi: 10.1186/s13613-021-00923-4, 34524562 PMC8441237

[43] Fujikawa K Koga T Honda T Uchida T Okamoto M Endo Y . Serial analysis of cytokine and chemokine profiles and viral load in severe fever with thrombocytopenia syndrome: case report and review of literature. Medicine (Baltimore). (2019) 98:e17571. doi: 10.1097/md.0000000000017571, 31626125 PMC6824633

[44] Nakamura S Iwanaga N Hara S Shimada S Kashima Y Hayasaka D . Viral load and inflammatory cytokine dynamics associated with the prognosis of severe fever with thrombocytopenia syndrome virus infection: an autopsy case. J Infect Chemother. (2019) 25:480–4. doi: 10.1016/j.jiac.2019.01.013, 30824300

[45] Shen L Han M Lou J Shen W Li S. Can antiviral drugs address the treatment challenges of severe fever with thrombocytopenia syndrome? - a systematic Meta-analysis. J Infect Dev Ctries. (2024) 18:1645–52. doi: 10.3855/jidc.19032, 39693154

