# Supplementary material for: The impact of different therapies on invasive pulmonary aspergillosis in patients with severe fever and thrombocytopenia syndrome: a systematic review and meta-analysis
Source: Front Public Health. 2026 Jul 8;14:1834363. doi: 10.3389/fpubh.2026.1834363 (PMC13388464; doi:10.3389/fpubh.2026.1834363)
Supplement: Supplementary file 1 [file Supplementary_file_1.docx]

**Supplementary materials**

**Figures and tables**


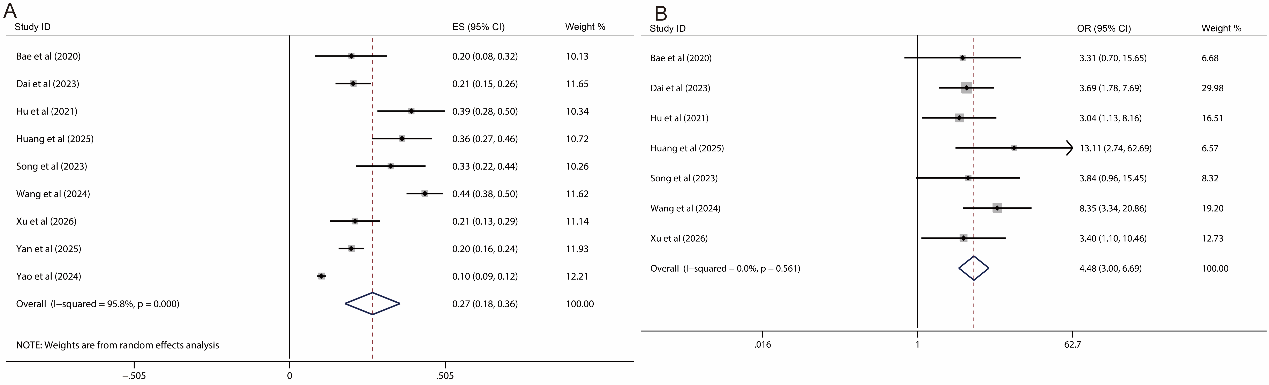


**Figure S1** Forest plot (excluding the study of Xu et al (2021)) showing the overall effect and 95% CI of SAPA incidence rate (A), mortality risk (B)


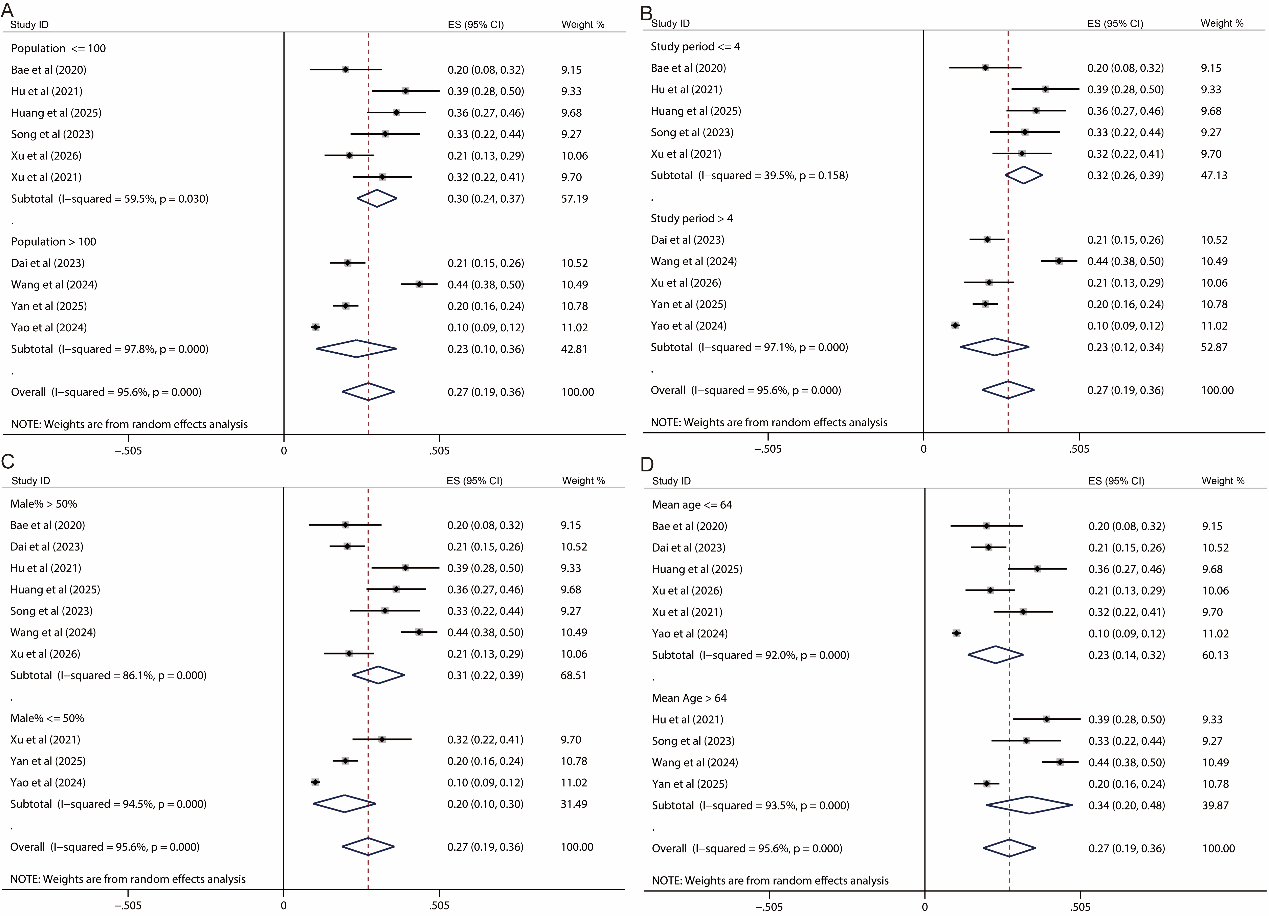


**Figure S2** Subgroup analysis of overall effect and 95%CI for SAPA incidence rate. A (population); B (study period); C (male %); D (mean age)


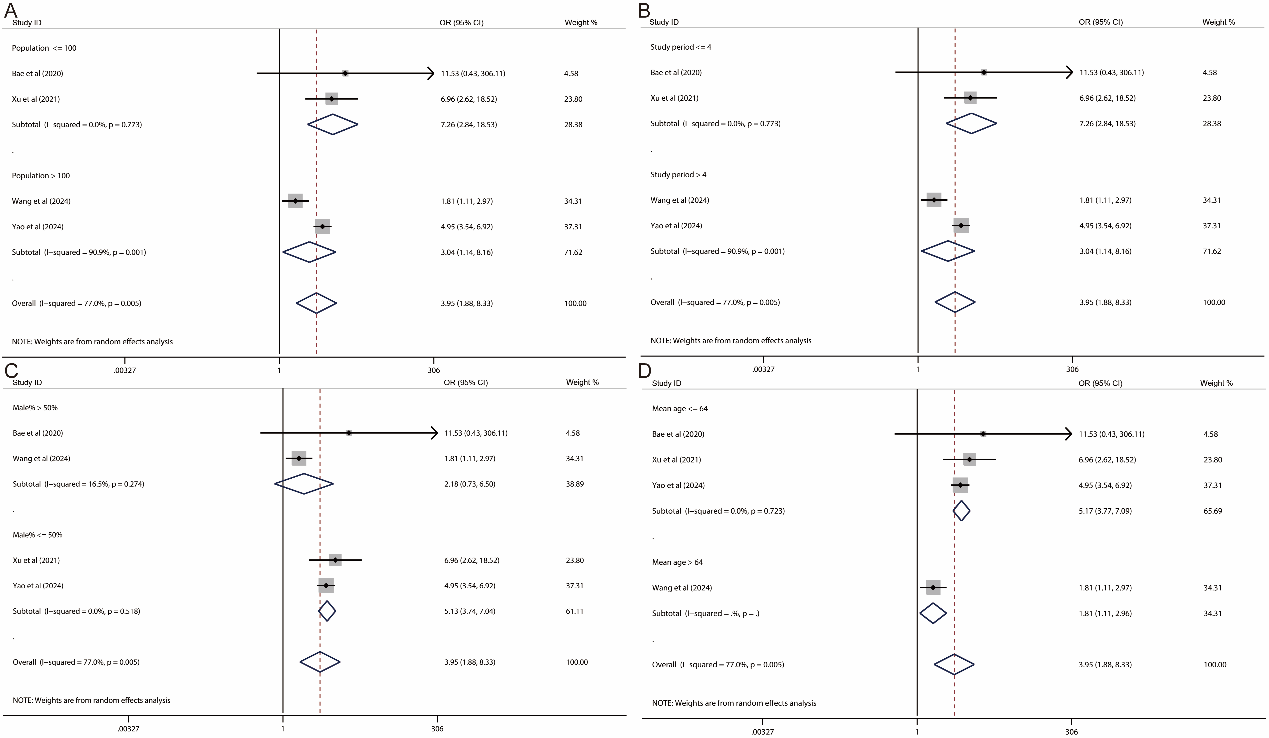


**Figure S3** Subgroup analysis of overall OR and 95%CI for IVIG therapy. A (population); B (study period); C (male %); D (mean age)

**Table S1** Search strategy in different databases.

| **Search terms for PubMed (n = 31), until 4 March 2026** |
| --- |
| #1 ("Severe Fever with Thrombocytopenia Syndrome" OR "SFTS" OR "SFTSV" OR "Dabie Banda virus") |
| #2 ("Invasive Pulmonary Aspergillosis" OR "IPA" OR "Aspergillus") |
| (#1 AND #2) |
| **Search terms for Web of Science (n =** 45**), until 4 March 2026** |
| #1 Topic = (Severe Fever with Thrombocytopenia Syndrome" OR "SFTS" OR "SFTSV" OR "Dabie Banda virus") |
| #2 Topic = ("Invasive Pulmonary Aspergillosis" OR "IPA" OR "Aspergillus") |
| (#1 AND #2) |
| **Search terms for Embase (n = 48), until 4 March 2026** |
| #1 'Severe Fever with Thrombocytopenia Syndrome':ti OR 'SFTS':ti OR SFTSV:ti OR Dabie Banda virus':ti |
| #2 'Invasive Pulmonary Aspergillosis':ti OR IPA *:ti OR Aspergillus *':ti |
| (#1 AND #2) |

**Table S2:** **PICOS framework**

| **PICO Directory** | **Details** |
| --- | --- |
| **Population (P)** | Patients of any age with laboratory‑confirmed SFTS, regardless of disease severity or baseline characteristics. |
| **Intervention/Exposure (I)** | For the incidence objective, the event of interest was the development of SAPA. For the mortality objective, the exposure was SAPA (proven or probable). For the therapeutic objective, the interventions were any therapies administered to SFTS patients that were evaluated for their effect on SAPA incidence. |
| **Comparator (C)** | For incidence, no comparator was necessary. For mortality, the comparator comprised SFTS patients without SAPA. For the therapy analyses, the comparators comprised SFTS patients who did not receive the corresponding therapy. |
| **Outcomes (O):** | The primary outcomes were the incidence of SAPA among SFTS patients and all‑cause mortality. The secondary outcome was the effects of different therapies on the SAPA rate. |
| **Study design (S)** | Eligible study designs included observational studies (retrospective and prospective cohort studies, case‑control studies) reporting original data. Reviews, corrigenda, editorials, and case reports were excluded. |

**Table S3:** NOS scores of included studies

| First author | NOS | Is the case definition adequate? | Representativeness of the cases | Selection of Controls | Definition of Controls | Comparability | Ascertainment of exposure | Same method of ascertainment for cases and controls | NOn-Response Rate |
| --- | --- | --- | --- | --- | --- | --- | --- | --- | --- |
| Bae et al (2020) | 6 | 1 | 1 | 1 | 0 | 1 | 1 | 0 | 1 |
| Dai et al (2023) | 8 | 1 | 1 | 1 | 1 | 1 | 1 | 1 | 0 |
| Hu et al (2021) | 7 | 1 | 1 | 1 | 1 | 1 | 1 | 1 | 0 |
| Huang et al (2025) | 8 | 1 | 1 | 1 | 1 | 1 | 1 | 1 | 0 |
| Song et al (2023) | 8 | 1 | 1 | 1 | 1 | 1 | 1 | 1 | 0 |
| Wang et al (2024) | 7 | 1 | 1 | 1 | 0 | 1 | 1 | 1 | 0 |
| Xu et al (2026) | 8 | 1 | 1 | 1 | 1 | 1 | 1 | 1 | 0 |
| Xu et al (2021) | 7 | 1 | 1 | 1 | 0 | 1 | 1 | 1 | 0 |
| Yan et al (2025) | 8 | 1 | 1 | 1 | 1 | 1 | 1 | 1 | 0 |
| Yao et al (2024) | 9 | 1 | 1 | 1 | 1 | 1 | 1 | 1 | 1 |

**Table S4** Begg’ test of the overall effect and 95% CI of different variables

| Variable | Begg’ test | | Egger’ test | |
| --- | --- | --- | --- | --- |
|  | z | *P* | t | *P* |
| SAPA incidence rate | 0.36 | 0.721 | 4.19 | 0.033 |
| Mortality risk | 0.87 | 0.386 | 0.74 | 0.486 |
| Corticosteroid therapy | 0.24 | 0.806 | 0.37 | 0.734 |
| IVIG therapy | 0.34 | 0.734 | 0.16 | 0.887 |
| Ribavirin therapy | 0.00 | 1.00 | 0.15 | 0.902 |
| Antibiotics therapy | 1.02 | 0.308 | -1.89 | 0.199 |
| Mechanical ventilation | 0.00 | 1.00 | -1.89 | 0.199 |

**Table S5** the 2×2 raw data of the included studies

| First author | SAPA/  non-SAPA | Deaths/  survivors | Corticosteroids therapy/no | IVIG  therapy/no | Ribavirin therapy/no | Antibiotics therapy/no | Mechanical ventilation |
| --- | --- | --- | --- | --- | --- | --- | --- |
| Bae et al (2020) | 9 | 4/5 | 6/4 | 1/8 | 2/7 | 9/0 | 9/0 |
|  | 36 | 7/29 | 5/31 | 0/36 | 9/27 | 32/4 | 5/31 |
| Dai et al (2023) | 39 | 21/18 | 30/9 | NR | NR | 26/13 | 15/24 |
|  | 150 | 36/114 | 68/82 | NR | NR | 12/138 | 15/135 |
| Hu et al (2021) | 30 | 16/14 | NR | NR | NR | 21/9 | NR |
|  | 46 | 8/38 | NR | NR | NR | 7/39 | NR |
| Huang et al (2025) | 35 | 12/23 | NR | NR | NR | NR | NR |
|  | 61 | 2/59 | NR | NR | NR | NR | NR |
| Song et al (2023) | 22 | 6/16 | NR | NR | NR | NR | NR |
|  | 45 | 4/41 | NR | NR | NR | NR | NR |
| Wang et al (2024) | 118 | 30/88 | 49/69 | 75/33 | NR | NR | NR |
|  | 151 | 6/147 | 38/113 | 84/67 | NR | NR | NR |
| Xu et al (2026) | 21 | 7/14 | NR | NR | NR | NR | NR |
|  | 78 | 10/68 | NR | NR | NR | NR | NR |
| Xu et al (2021) | 29 | 11/18 | 8/21 | 20/9 | 29/0 | NR | NR |
|  | 62 | 7/55 | 10/52 | 15/47 | 55/7 | NR | NR |
| Yan et al (2025) | 72 | NR | NR | NR | NR | NR | NR |
|  | 288 | NR | NR | NR | NR | NR | NR |
| Yao et al (2024) | 169 | NR | 74/95 | 108/61 | 118/51 | 169/0 | 36/133 |
|  | 1481 | NR | 273/1280 | 390/1091 | 902/579 | 1430/51 | 61/1420 |
